# A Grateful Patient

**Published:** 1920-10-23

**Authors:** 


### A Grateful Patient.

It is pleasant to read of an engine-driver's fortune of £3,255 net personalty, which was left by Mr. George Read, of Kentish Town, who died at Hanwell on March 15 last. The will of the deceased provided not only for his housekeeper and her two daughters, but in addition to bequests of £100 each to the Foundling Hospital and Dr .Barnardo's Homes, he left £10 to his doctor.

